# Sperm-derived histones contribute to zygotic chromatin in humans

**DOI:** 10.1186/1471-213X-8-34

**Published:** 2008-03-31

**Authors:** Godfried W van der Heijden, Liliana Ramos, Esther B Baart, Ilse M van den Berg, Alwin AHA Derijck, Johan van der Vlag, Elena Martini, Peter de Boer

**Affiliations:** 1 Department of Obstetrics and Gynaecology, Radboud University Nijmegen Medical Centre, P.O. Box 9101, 6500 HB Nijmegen, The Netherlands; 2 Division of Reproductive Medicine, Department of Obstetrics and Gynaecology, Erasmus MC, University Medical Center, Gravendijkwal 230, 3015 CE Rotterdam, The Netherlands; 3 Nephrology Research Laboratory, Nijmegen Centre for Molecular Life Sciences, Division of Nephrology, Radboud University Nijmegen Medical Centre, P.O. Box 9101, 6500 HB, Nijmegen, The Netherlands; 4 Carnegie Institution of Washington, Department of Embryology, 3520 San Martin Drive, Baltimore, MD 21218, USA; 5 University Medical Center Utrecht, Department of Reproductive Medicine and Gynaecology,, Heidelberglaan 100, 3584 CX Utrecht, The Netherlands; 6 University Medical Center Utrecht, Department of Pharmacology and Anatomy, Rudolf Magnus Institute of Neuroscience, Universiteitsweg 100, 3584 CG, Utrecht, The Netherlands

## Abstract

**Background:**

about 15% to 30% of the DNA in human sperm is packed in nucleosomes and transmission of this fraction to the embryo potentially serves as a mechanism to facilitate paternal epigenetic programs during embryonic development. However, hitherto it has not been established whether these nucleosomes are removed like the protamines or indeed contribute to paternal zygotic chromatin, thereby potentially contributing to the epigenome of the embryo.

**Results:**

to clarify the fate of sperm-derived nucleosomes we have used the deposition characteristics of histone H3 variants from which follows that H3 replication variants present in zygotic paternal chromatin prior to S-phase originate from sperm. We have performed heterologous ICSI by injecting human sperm into mouse oocytes. Probing these zygotes with an antibody highly specific for the H3.1/H3.2 replication variants showed a clear signal in the decondensed human sperm chromatin prior to S-phase. In addition, staining of human multipronuclear zygotes also showed the H3.1/H3.2 replication variants in paternal chromatin prior to DNA replication.

**Conclusion:**

these findings reveal that sperm-derived nucleosomal chromatin contributes to paternal zygotic chromatin, potentially serving as a template for replication, when epigenetic information can be copied. Hence, the execution of epigenetic programs originating from transmitted paternal chromatin during subsequent embryonic development is a logical consequence of this observation.

## Background

A hallmark of spermiogenesis is the transformation of the chromatin of the germ cell. In elongating spermatids nucleosomes are replaced by transition proteins, which are subsequently replaced by protamines. The protamine-based chromatin allows a dense packing of the DNA, which facilitates its protection and transportation to the oocyte (see for a review [1]). The replacement of nucleosomes by protamines is frequently observed throughout the animal kingdom [2], though the degree of this chromatin substitution varies among species. Protamination in mouse and boar is almost complete and only an estimated 1% of the total DNA in mouse sperm cells remains nucleosome bound, whereas in human sperm this is estimated to be about 15% [3-5]. Characterisation of the histones in human sperm identified H2A, H2AX, H2AZ, H2B, H3.1, H3.3, CenH3 and H4 [4,6].

Analysis of the preferential chromatin conformation (nucleosome and/or protamine based) of several genes and structural elements in human sperm showed limited differences between sperm of one individual, but also between sperm of different individuals [7-9]. This sequence-specific chromatin conformation has been suggested to facilitate transcriptional activation of paternal genes in early embryogenesis and enable the three-dimensional organization of the sperm nucleus [6-8]. However, whether sperm- derived nucleosomes contribute to zygotic chromatin, a necessity to enable such epigenetic programs, or are removed during the extensive chromatin remodelling occurring after gamete fusion [10-12] has hitherto not been established. Roles for the maintenance of imprinting, as speculated by [13] has recently been illustrated in Arabidopsis, where, contrary to the zygote proper, a specific paternal H3.3 isoform was maintained in the endosperm, the plant tissue where imprinting pays a role [14]

Therefore, we set out to detect histones of sperm origin in paternal zygotic chromatin. Since deposition of maternally derived histones takes place immediately after gamete fusion [10], sperm-derived histones, if retained, become indistinguishable from maternal ones. To circumvent this problem we used the difference in deposition characteristics of the histone H3 variants. The histones H3.1/H3.2 (the replication variants) are assembled into nucleosomes when DNA replication occurs, in contrast to histone H3.3, which is only incorporated outside the context of DNA replication [15]. Hence, shedding of protamines after sperm entry is followed by deposition of the H3.3 – H4 dimer chaperoned by Hira [10,16]. Deposition of maternal H3.1/H3.2 starts at the onset of zygotic S-phase, which commences approximately 8 hours after insemination in human zygotes [17]. Therefore, all H3.1/H3.2 present in the paternal chromatin prior to S-phase must originate from the male germ line.

## Methods

### Sperm decondensation in vitro

Sperm head decondensation was achieved by incubation of sperm samples in PBS containing 0.2% Triton X-100, 100 IU heparin (Leo Laboratories) and 2.5 mM DTT at room temperature [18,19]. In order to obtain more than 80% decondensed heads per sample, incubation time varied between 10 to 15 minutes. The decondensation process was stopped by immersing the glass slides in 4% paraformaldehyde (PFA) for 15 minutes. Slides were then washed twice in PBS and allowed to dry.

### Preparation of Cryo-preserved human sperm for heterologous ICSI

Cryo-straws containing 500 μl sperm suspension were thawed at room temperature and 1000 μl HTF-HEPES was added and gently mixed. The content was transferred to an eppendorf vial and centrifuged for 5 minutes at 500 xg. Subsequently the supernatant was discarded and the pellet was gently dissolved in HTF-HEPES and kept at room temperature.

### Preparation of mouse oocytes for heterologous ICSI

*B6D2 *F1 females (Charles River, Sulzfeld, Germany) were used as oocyte donors and were kept in an adjusted light schedule, set at 9.00 am – 9.00 pm. Superovulation was induced by i.p. injection of 7.5 IU pregnant mare's serum gonadotrophin (PMSG, Intervet, Boxmeer, The Netherlands) around 9 pm, followed by 7.5 IU hCG (Intervet) after 48 h. Oocytes were harvested from the oviducts 13 h after administration of hCG and stored without cumulus cells at 37°C for up to 5 h in complemented Mem-α [20].

### Heterologous intra cytoplasmic sperm injection

Microinjection was performed as described in [21] and [20] with some adaptations. The temperature in the injection droplet was kept at 24°C. For each ICSI experiment cryo-preserved sperm samples were freshly prepared (see above). Each injection round a sperm aliquot was transferred to medium containing 12% polyvinyl pyrrolidone. Sperm were selected for normal morphology and motility. After injection, oocytes were kept on the injection platform for 5 minutes, then gradually warmed to 37°C and placed in culture medium at 37°C, 5% CO2 in air [20].

### Immobilization, fixation and immunofluorescence staining of mouse zygotes

Prior to fixation of the zygotes the zona pellucida was removed with acidic tyrode (pH 2.5) containing 1% BSA. Thereafter, cells were immobilised in a fibrin clot [22]. Fibrinogen was obtained from Calbiochem, cat. nr. 341573; Thrombin was obtained from Sigma, cat. nr. T-6634. Cells were fixed in 2% PFA, 0.15% Triton X-100 for 30 minutes, followed by incubation in ice-cold methanol for 10 minutes. Immunofluorescence was performed as described previously [10].

### Antibodies

The monoclonal antibody #34 was used at a dilution of 1:1500 to detect H3.1/H3.2 (for characterisation see [10]). Additional proof of antibody specificity was obtained by probing nuclear extracts of tagged H3 isoform transgenic mice on western blots with ab #34 [23]. Polyclonal rabbit Pan-H3 (Abcam ab1791) was used at a dilution of 1:500. To unmask the epitope for the pan-H3 antibody slides were first incubated in 4 M HCl for 6 minutes prior to blocking, after which slides were extensively washed in PBS. Primary antibodies were detected by Molecular Probes A11001 fluor 488 goat anti-mouse IgG (H+L) and A11012 fluor 594 goat anti-rabbit IgG (H+L). Both were used in a 1:500 dilution.

### Collection and fixation of human polypronuclear zygotes

Polypronuclear zygotes, i.e. zygotes that show three or more pronuclei after insemination instead of the expected two, are considered to be non-diploid and are therefore never transferred to the uterus in a human IVF setting. Polypronuclear zygotes used in this study were obtained from couples undergoing routine IVF procedures at the Erasmus MC in the period between June and September 2005. This study was approved by the Dutch Central Committee on Research involving Human Subjects (CCMO) and the local ethics review committee of the Erasmus university medical center. Human zygotes were produced by conventional in vitro fertilization (IVF) or after intracytoplasmic sperm injection (ICSI) [24,25]. After IVF, remaining cumulus cells and sperm cells were removed from the oocytes by gentle aspiration ~7 hrs after insemination and oocytes were moved to fresh drops of culture medium. All oocytes after both IVF and ICSI were checked hourly until 10 hrs past insemination for the appearance of pronuclei. To ensure that zygotes were fixed before the onset of S-phase, they were collected as soon as more then two clearly discernible pronuclei were observed. Before fixation, zona pellucida were removed from zygotes by pronase treatment (0.5% in HEPES-buffered medium). Immobilization and fixation occurred as described above. After washing in PBS, fixed zygotes were either stored at 4°C in PBS containing 10% normal goat serum and 0.05% NaN_3 _or frozen in culture medium containing 1.5 M DMSO.

## Results

### Presence of histone H3 in human sperm

**Figure 1:**
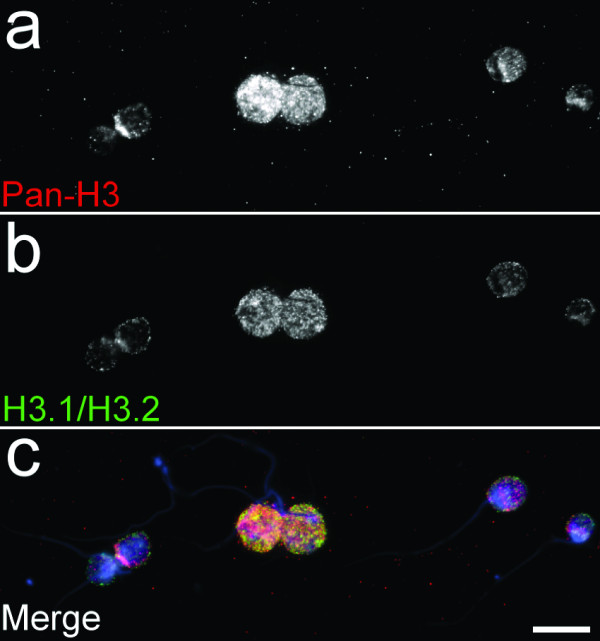
**Presence of histone H3 variants in human sperm**. Human sperm, treated with heparin to induce chromatin decondensation, stained with a pan-H3 antibody (**a**), which recognizes all histone H3 variants (H3.1, H3.2 and H3.3) or a H3.1/H3.2-specific antibody (**b**) revealed for both antibodies a diffuse, global staining. The merge (**c**) with DAPI, which labels DNA, shows the nuclear localisation of the histones.

Both the replacement and replication variants are detected in sperm by HPLC analysis [4]. To visualize histone H3 in sperm by immunofluorescence, we decondensed the cells by incubation in a mixture containing heparin and DTT. The dense structure of sperm chromatin does not allow antibody penetration without this treatment. Staining of decondensed sperm with the pan-H3 and H3.1/H3.2 antibodies showed a relatively homogeneous structure revealing signal for both (see Fig. 1).

### Localisation of replication H3 variants in human paternal chromatin after heterologous ICSI

**Figure 2:**
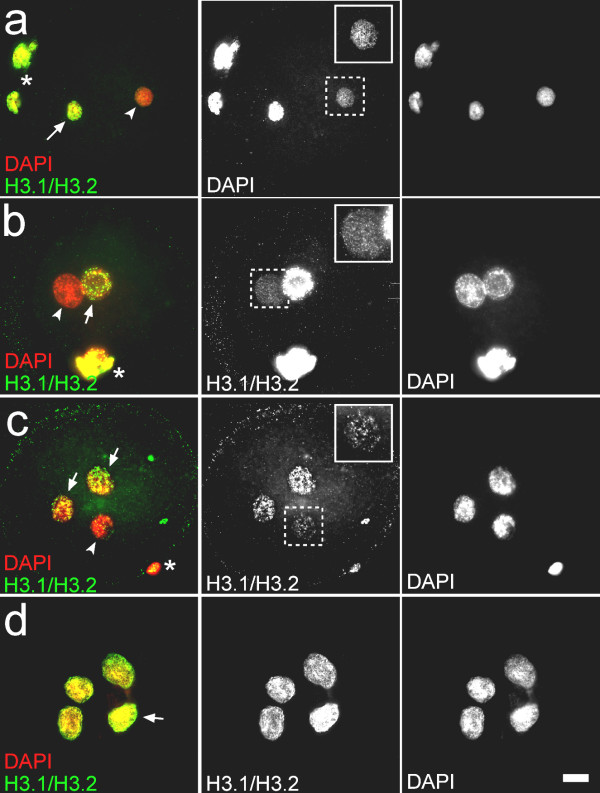
**Presence of histone H3.1/H3.2 in paternal chromatin derived from human sperm in mouse and human oocytes**. Zygotes obtained after heterologous ICSI (**a, b**) and human multipronuclear zygotes acquired after artificial fertilisation (**c, d**) stained for H3.1/H3.2 (middle column) and DAPI, which labels DNA (right column); merges are depicted in the left column. Zygotes were obtained after heterologous ICSI and fixed respectively 60 minutes (**a**) and 150 minutes (**b**) after injection of human sperm. Arrow indicates maternal chromatin, arrowhead paternal chromatin and asterisk the polar bodies.**c. **Human tripronuclear zygote fixed 7 hours after performing ICSI. Tripronuclear zygotes derived from ICSI are likely to be a consequence of a failure in second polar body extrusion. Similar to maternal mouse chromatin, these pronuclei exhibit clear H3.1/H3.2 staining (indicated by arrows). H3.1/H3.2 is also present in the paternal pronuclei (indicated by arrowhead) though the obtained signal is lower. Asterisk indicates polar body. **d. **Human multipronuclear zygote fixed 22 hours after insemination with human sperm. Multipronuclear zygotes derived from IVF are likely formed by fusion of multiple sperm with an oocyte. The more intensely stained pronucleus (arrow) most likely represents the female nucleus since H3.1/H3.2 levels are higher in maternal chromatin [10].

To find out whether human sperm derived histones are retained in paternal chromatin after decondensation, we determined the presence of H3 replication variants in paternal chromatin in zygotic G1. This signal will not be obscured by de novo nucleosome assembly from the oocyte. Sperm from three fertile donors was used for injection in mouse oocytes. After injection, zygotes were incubated for different periods of time (75 to 370 min) prior to fixation to allow detection of H3.1/H3.2 at various stages of G1 [10]. At all stages we observed a prominent H3.1/H3.2 staining of maternal chromatin and a minor but clear staining of the paternal chromatin (see Fig. 2a,b; n = 60). Localisation of H3.1/H3.2 was found throughout the male chromatin, not concentrated in specific regions, again revealing a certain structure. Due to the increase of paternal pronuclear size at G1 in time, signals became diluted later on. The visualized H3.1/H3.2 fraction in paternal chromatin (Fig. 2a–c) is an underrepresentation of the total amount of H3 transmitted by the male gamete since the H3 replacement variant (H3.3) is also present in sperm [4] (Fig 1).

### Localisation of replication H3 variants in paternal chromatin in human polypronuclear zygotes

To determine whether the H3 replication variants are also present in zygotic paternal human chromatin in a homologous setting, we localised H3.1/H3.2 in abnormally fertilized human oocytes, produced by IVF. From seven hours after insemination, appearance of the pronuclei was visually assessed every hour. Zygotes in which 3 or more pronuclei were observed were collected and fixed. Histone H3.1/H3.2 staining revealed a localisation reminiscent to that of the heterologous zygotes. Maternal chromatin clearly contained histone H3.1/H3.2, whereas the sperm-derived chromatin according to expectation exhibited a less intense staining (see Fig 2c; n = 8). Zygotes after S-phase had a clear increase in pronuclear histone H3.1/H3.2 levels, confirming the replication kinetics of these H3 species (see Fig 2d; n = 9).

## Discussion

The relative abundance of nucleosomal chromatin and the presence of modified histones in human sperm [4] potentially allows a protein-based epigenetic program [13]. It has been suggested that genes that are contained in nucleosomal chromatin undergo earlier transcriptional activation as opposed to genes in protamine-based chromatin [7]. This implies a function for the dual nucleosome/protamine chromatin structure in regulation of gene expression in the early embryo [1]. Furthermore, it has also been hypothesized that the cue which triggers inactivation of the paternally derived X chromosome (X_p_) after gamete fusion is contained in the transmitted nucleosomal chromatin of the X_p _[26]. A requirement for such paternal-derived epigenetic program to operate in the zygote is that sperm derived nucleosomes must be retained in paternal chromatin. After gamete fusion, sperm chromatin is subjected to intense remodelling and the protamines but possibly also the nucleosomes are removed from the DNA. Previously, we have shown that in mouse, modified nucleosomes present in sperm are transmitted to the zygote [18]. Here, we demonstrate that in human sperm, the H3 replication variants contribute to paternal chromatin in the zygote (see Fig. 2). Since histone H3 forms the nucleosome complex with the other histone proteins, it is conceivable that also these other histones are retained in the paternal chromatin in the zygote. Recent work showed that under in vitro conditions H3.1/3.2 containing nucleosomes are much more stable than H3.3 containing nucleosomes [27]. This difference might explain in part the association of H3.3 with actively transcribed genes and H3.1/H3.2 with silent regions [28]. Paternal sequences wrapped around H3.1/H3.2 containing nucleosomes could be poised to transcriptional inactivity and initiate self-propagation of this state at the service of structural chromosomal elements like centric heterochromatin ((human sperm nuclei [6]; bull sperm nuclei [29]; mouse sperm and zygote [18]); Arabidopsis zygote and endosperm nuclei [14]). However, also sperm derived H3.3-containing nucleosomes are likely to contribute to the paternal chromatin. A recent study with transplantation experiments in Xenopus embryos suggested that histone H3.3 has a role in the memory of transcription states, also in the absence of actual gene transcription [32].

An estimated 15% of the genome in human sperm remains in a nucleosomal context [3-5]. The epigenetic potential by incorporation of these sperm derived nucleosomes into the paternal zygotic chromatin is obvious [13]. Future research, however, is needed to establish whether such programs exist.

The treatment of infertility via artificial reproduction enables men with hampered spermatogenesis to sire offspring. Recent studies have indicated a decrease in the protamine/nucleosome ratio when comparing sperm from infertile men to fertile men [19,30]. Also the kinetics of paternal CpG demethylation could be affected by the presence of nucleosomes [31,32].

## Conclusion

The use of a H3.1/H3.2 specific antibody enabled demonstration of paternal histones in pre-S-phase pronuclear human zygotes and zygotes produced by human sperm and mouse oocytes. This could have consequences for early developmental processes via enabling downstream effects of sperm derived nucleosomal chromatin that contains variants and/or carries histone modifications. A higher nucleosomal content in sperm as is now found in human oligospermia would then lead to expanded epigenetic transmission.

## Authors' contributions

LR, GWvdH, and AAHAD executed the heterologous ICSI experiments, EBB and IMvdB executed the human multipronuclear experiments. EM supervised the experiments with human zygotes. GWvdH was responsible for the first manuscript. JvdV characterized the pivotal antibody and assisted in manuscript preparation. PdB supervised the project. All authors read and approved the final mauscript.
